# Supplementary material for: Silver Nimesulide Complex in Bacterial Cellulose Membranes as an Innovative Therapeutic Method for Topical Treatment of Skin Squamous Cell Carcinoma
Source: Pharmaceutics. 2022 Feb 21;14(2):462. doi: 10.3390/pharmaceutics14020462 (PMC8877292; doi:10.3390/pharmaceutics14020462)
Supplement: Supplementary file 1 [file pharmaceutics-14-00462-s001.zip › pharmaceutics-1567576-supplementary.pdf]

pharmaceutics MDPI

Supplementary Materials: Silver Nimesulide Complex in Bacterial Cellulose Membranes as an Innovative Therapeutic Method for Topical Treatment of Skin Squamous Cell Carcinoma

Tuany Zambroti Candido, Raphael Enoque Ferraz de Paiva, Mariana Cecchetto Figueiredo, Lilian de Oliveira Coser, Silmara Cristina Lazarini Frajácomo, Camilla Abbehausen, Izilda Aparecida Cardinalli, Wilton Rogerio Lustri, João Ernesto Carvalho, Ana Lucia Tasca Gois Ruiz, Pedro Paulo Corbi and Carmen Silvia Passos Lima

Table S1. Influence of AgNMS on phosphatidilserine residues exposure and multicaspase activation on FaDu cells after 24 and 48 h exposure.

Cell subpopulation (%)		Control[a]	AgNMS[b]			
			10 μM		100 μM	
			24 h	48 h	24 h	48 h
Phosphatidilserine residues exposure[c]	Q1	36.3 ± 20.3	54.9 ± 33.2	40.7 ± 4.2	20.1 ± 27.5	16.9 ± 23.9
	Q2	0.10 ± 0.08	0.5 ± 0.5	0.01 ± 0.01	0.01 ± 0.01	0.0 ± 0.0
	Q3	14.2 ± 6.8	9.4 ± 6.4	8.9 ± 2.3	49.0 ± 33.4	52.7 ± 21.9
	Q4	49.5 ± 13.6	35.4 ± 27.4	50.4 ± 2.0	31.0 ± 5.9	30.5 ± 1.9
Multicaspaseactivation[d]	Q1	0.7 ± 0.1	0.07 ± 0.09	1.5 ± 0.4	0.02 ± 0.02	0.3 ± 0.3
	Q2	0.21 ± 0.09	0.04 ± 0.04	0.16 ± 0.08	0.0 ± 0.0	0.3 ± 0.4
	Q3	18.3 ± 9.6	32.2 ± 14.1	8.6 ± 1.7	37.2 ± 6.7**	25.5 ± 11.2***
	Q4	80.8 ± 9.8	67.8 ± 11.9	89.7 ± 2.1	62.9 ± 6.7**	73.9 ± 10.4**

Results expressed as mean (%) ± standard deviation from duplicates of one independent experiment. a) control = untreated cells. b) AgNMS = silver nimesulide complex at 10 and 100 μM, after 24 and 48 h exposure.c) Cell subpopulation (%) for phosphatidilserine residue exposure: Q1 = anexin 5-PE (-)/7-AAD (-); Q2 = anexin 5-PE (+)/7-AAD (-); Q3 = anexin 5-PE (+)/7-AAD (+); Q4 = anexin 5-PE (-)/7-AAD (+). d) Cell subpopulation (%) for multicaspase activation: Q1 = FAM (-)/7-AAD (-); Q2 = FAM (+)/7-AAD (-); Q3 = FAM (+)/7-AAD (+); Q4 = FAM (-)/7-AAD (+); FAM: carboxyfluorescein fluorochrome. Statistical analysis: two-way ANOVA followed by Tukey's test (*p < 0.05, **p < 0.01, ***p < 0.001 comparing to control group).

Table S2. Body weight evolution of mice during all experimental weeks.

Week	Group 1	Group 2	Group 3	Group 4
−1	25.9 ± 1.3	26.0 ± 0.8	23.6 ± 0.5	25.1 ± 0.8
0	27.4 ± 1.1	27.5 ± 0.6	26.1 ± 0.7	26.9 ± 1.1
1	29.1 ± 0.5	28.1 ± 0.3	26.9 ± 1.0	27.5 ± 1.1
2	30.0 ± 0.4	28.6 ± 0.4	28.0 ± 1.0	28.8 ± 1.2
3	30.8 ± 0.4	29.7 ± 0.6	29.0 ± 0.9	30.1 ± 1.2
4	31.5 ± 0.5	30.4 ± 0.4	29.3 ± 0.9	31.0 ± 1.2
5	31.5 ± 1.0	30.4 ± 0.6	29.3 ± 1.1	30.6 ± 1.5
6	32.4 ± 0.3	31.1 ± 0.7	30.1 ± 1.1	31.4 ± 1.5
7	33.2 ± 0.5	32.1 ± 1.1	30.7 ± 1.2	31.9 ± 10.4
8	32.7 ± 0.3	31.4 ± 0.9	31.0 ± 3.5	32.2 ± 3.4
9	33.4 ± 0.8	31.9 ± 0.8	29.6 ± 1.1	31.4 ± 1.5
10	33.2 ± 0.6	32.3 ± 0.5	31.6 ± 1.4	32.9 ± 1.7
11	33.4 ± 0.4	31.9 ± 0.7	31.3 ± 1.6	32.4 ± 1.6
12	33.9 ± 0.3	32.7 ± 0.4	31.7 ± 1.6	32.9 ± 1.9
13	33.3 ± 0.7	32.2 ± 0.6	31.0 ± 1.4	32.0 ± 2.1
14	34.8 ± 0.4	33.0 ± 0.5	32.1 ± 1.4	32.7 ± 2.0
15	34.9 ± 0.9	33.6 ± 0.3	32.3 ± 1.6	33.0 ± 2.0

16	34.4 ± 0.5	33.1 ± 0.9	32.1 ± 1.4	32.6 ± 1.7
17	34.7 ± 0.0	32.8 ± 0.1	32.0 ± 1.4	33.0 ± 1.7
18	34.4 ± 0.3	33.1 ± 0.6	32.1 ± 1.1	32.6 ± 1.6
19	34.7 ± 0.1	32.8 ± 0.2	32.0 ± 1.6	33.0 ± 1.8
20	34.7 ± 0.4	32.9 ± 0.9	32.1 ± 1.7	33.1 ± 1.8
21	35.1 ± 0.4	33.8 ± 0.5	32.9 ± 1.5	33.4 ± 1.9
22	34.3 ± 0.0	33.3 ± 0.0	32.1 ± 1.5	32.9 ± 2.0
23	34.3 ± 3.8	33.3 ± 1.2	32.2 ± 0.8	32.9 ± 1.9
24	32.7 ± 3.8	30.3 ± 1.5	28.7 ± 2.2	29.8 ± 2.1
25	33.2 ± 1.6	32.3 ± 0.9	31.5 ± 1.4	31.8 ± 3.1
26	33.1 ± 3.3	32.5 ± 0.6	31.6 ± 5.1	33.1 ± 4.4
27	34.2 ± 3.3	33.2 ± 0.6	33.1 ± 5.1	32.8 ± 4.4

Results expressed as mean (g) ± standard deviation (n = 20 animals/group, G2 and G4; n = 14 animals/group, G1 and G3) of mice (maleBalb/c) body weight. Experimental weeks: S-1 = 7,12-dimethylbenzanthracene (DMBA) treatment; S0 – S20 = 12-o-tetradecanoyl-phorbol-13-acetate(TPA) treatment; S21–S23 = Bacterial cellulose membrane (BCM) device treatment; S24–S27 = recovering time. Experimental groups: G1 = unloaded BCM device without skin cancer induction; G2 = unloaded BCM device with skin cancer induction; G3 = BCM with silver nimesulide complex (AgNMS@BCM) device without skin cancer induction; G4 = AgNMS@BCM device with skin cancer induction.

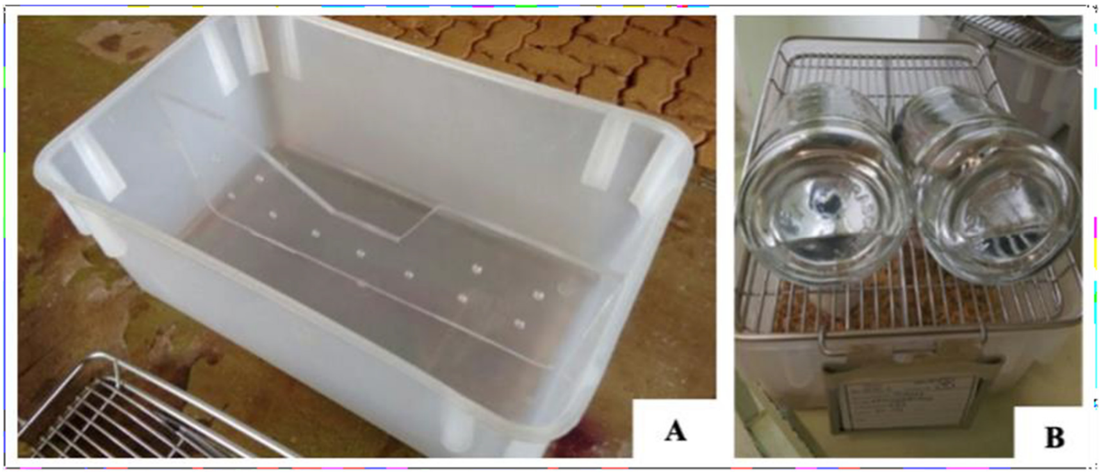

Figure S1. Polyethylene boxes (matte white) showing the transparent acrylic wall (**A**) used to allow visual and olfactory contact between mice without physical contact (**B**).

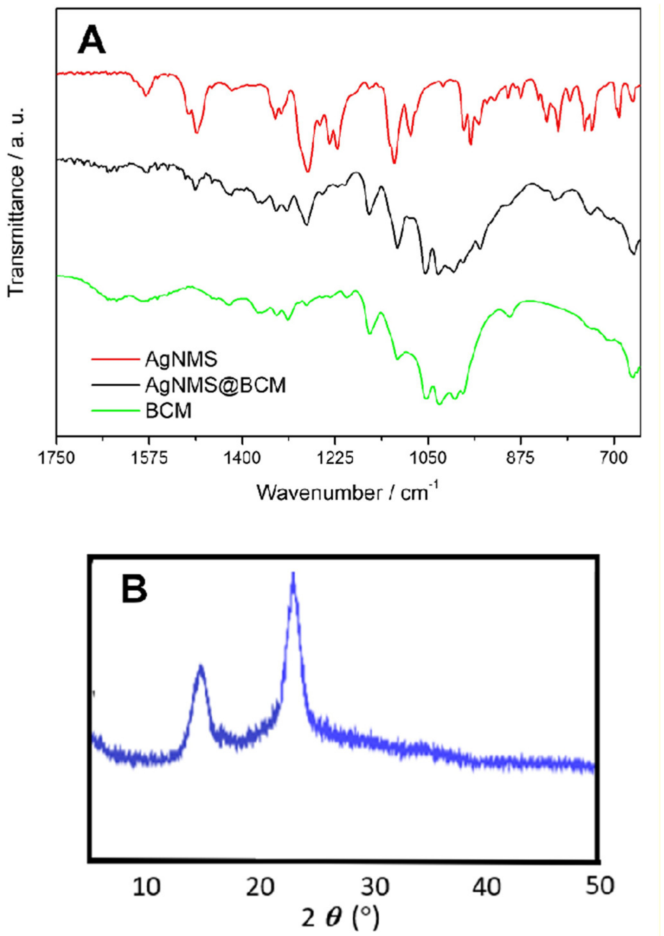

Figure S2. Bacterial cellulose membrane (BCM) characterized by Fourier transform infrared (FTIR) spectra **(A)** and X-ray diffraction (XRD) (**B**) The XDR pattern showed the typical profile and crystallinity degree of BCM. The main diffraction peaks were found at 2θ 14.7, 16.7 and 22.7, and assigned to diffraction planes (101), (101) and (002), respectively.

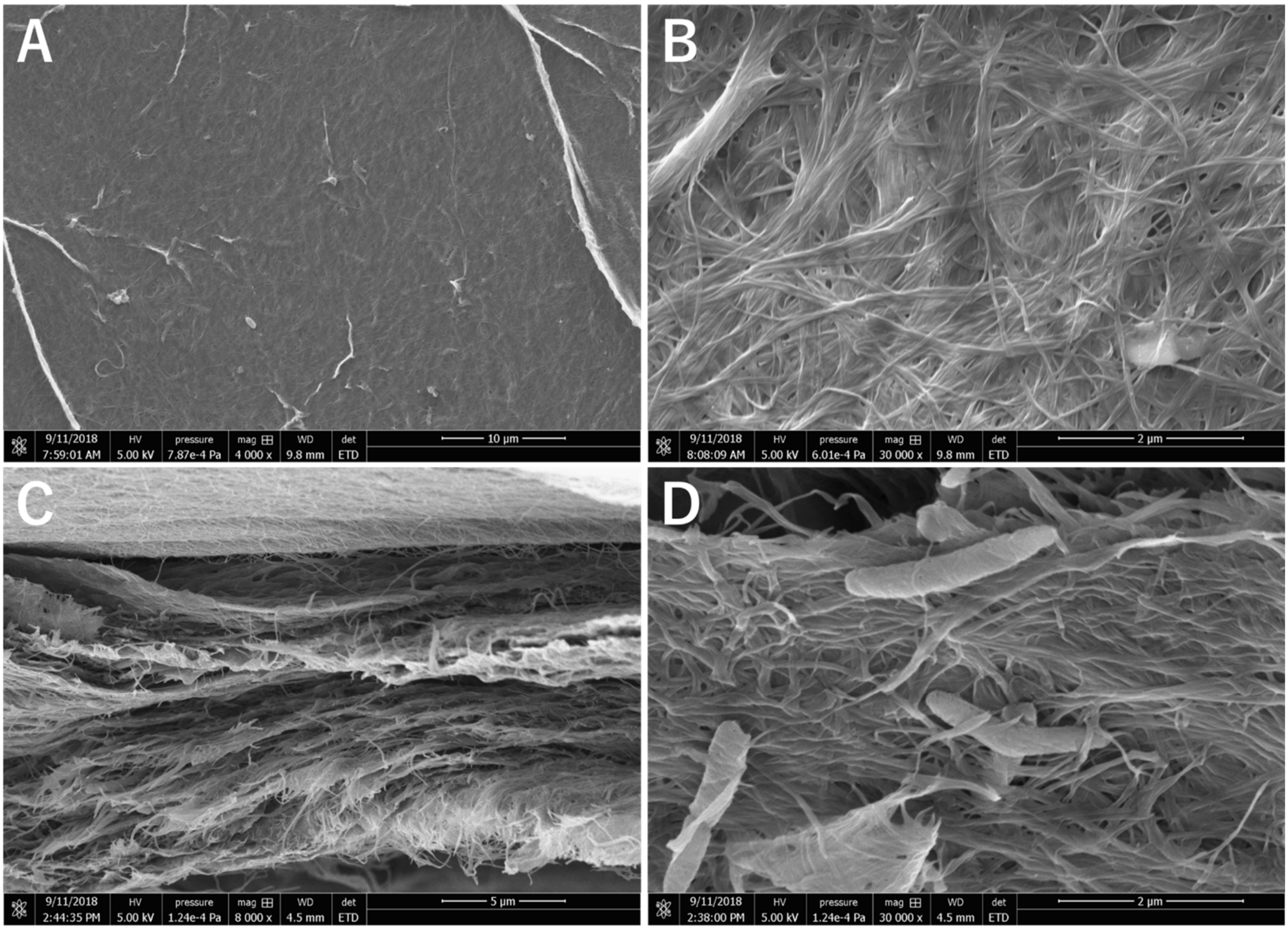

Figure S3. Microscopic characteristics of the pristine bacterial cellulose membrane. Overview (**A**) and finer details (**B**) of the surface. The cryofracture shows the internal network of bacterial cellulose fibers (**C**) and at higher magnification the bacterial cells (**D**) can also be observed.

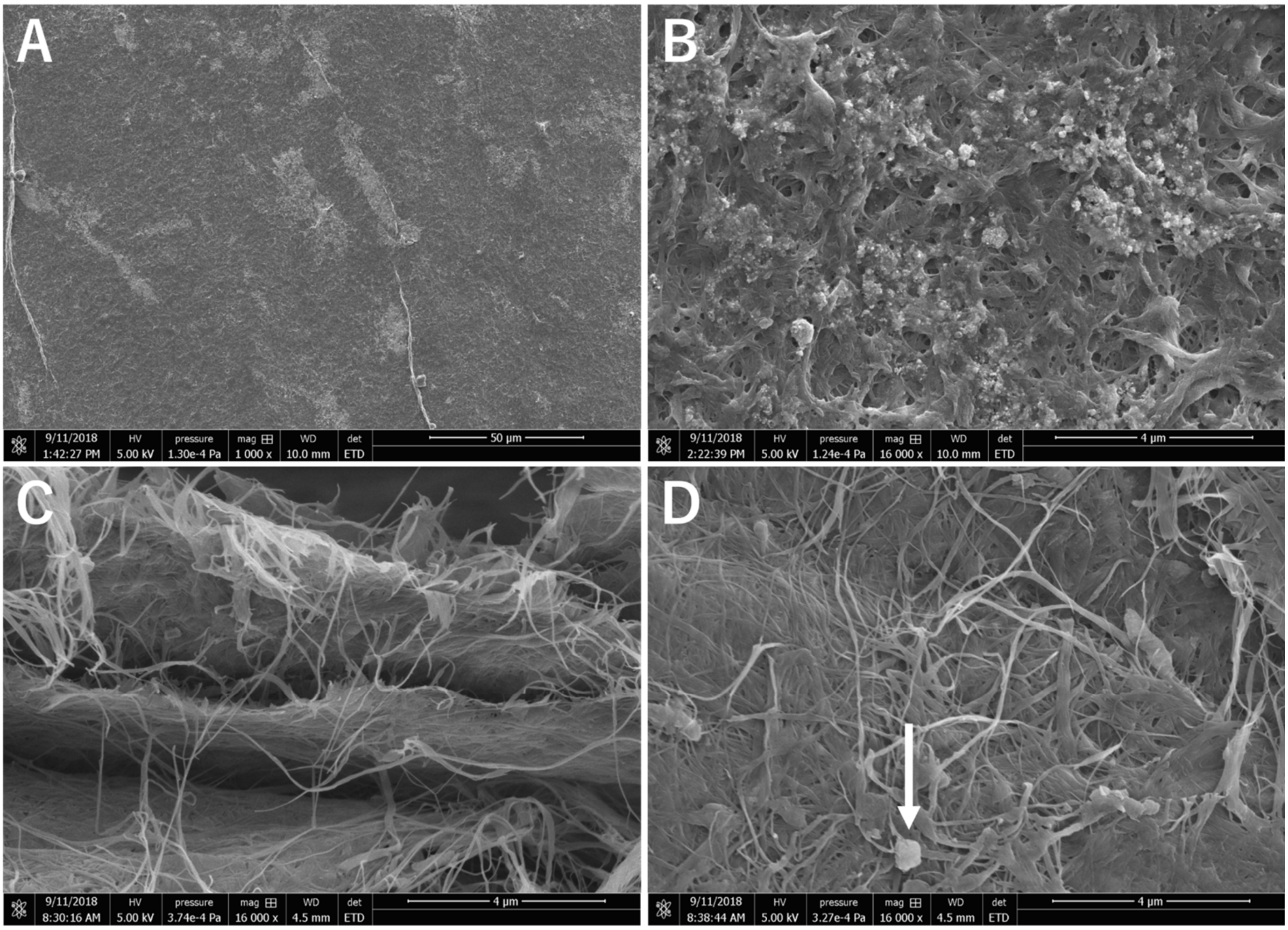

Figure S4. Microscopic characteristics of the AgNMS@BCM material. Overview (**A**) and finer details (**B**) of the surface, demonstrating the aggregates of AgNMS. The cryofracture (**C**,**D**) shows that the AgNMS aggregates can also be seen inside the material as highlighted by the white arrow.

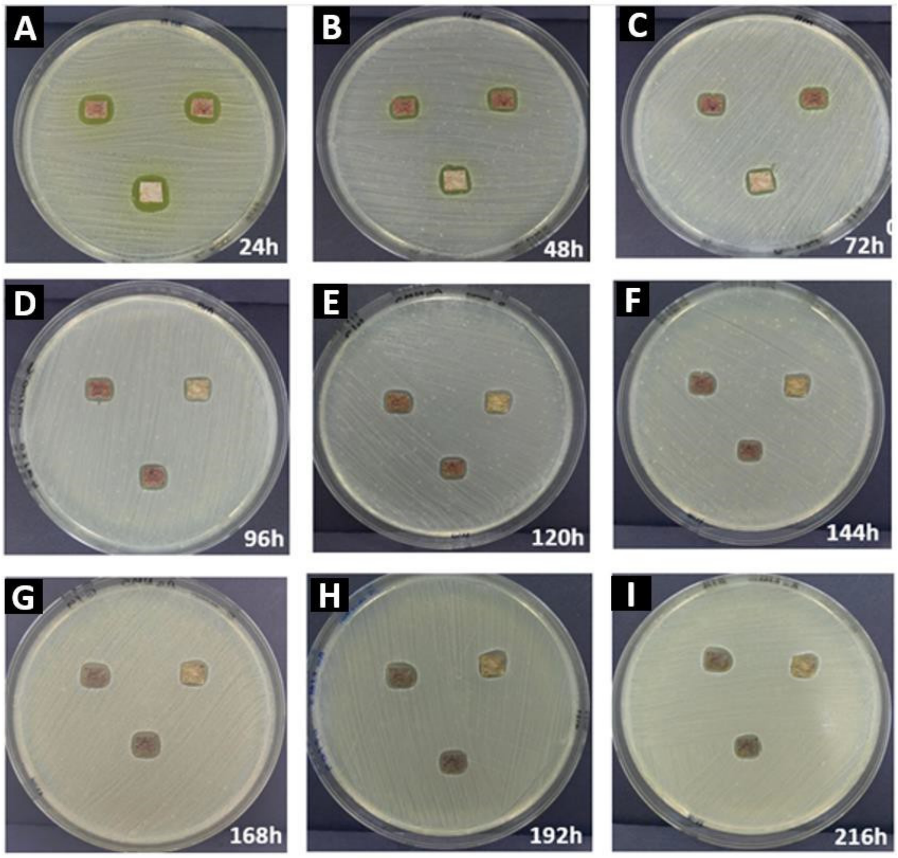

Exposure time (h)[a]	Growth inhibition halo(mm)[b]
24	15.0 ± 0.10
48	14.6 ± 0.15
72	14.6 ± 0.25
96	13.6 ± 0.20
120	13.0 ± 0.20
144	12.6 ± 0.25
168	12.6 ± 0.30
192	12.3 ± 0.35
216	12.0 ± 0.15

Figure S5. Antibacterial evaluation demonstrating the sustained release of the AgNMS complex from bac cellulose membrane (BCM).a) Discs of 1 cm^2 (in triplicate) of BCM loaded with AgNMS complex (1 mg c AgNMS@BCM.Every 24h, the discs were transferred to a new Mueller Hinton agar plate immediately after S *ylococcus aureus* ATCC 25923 inoculum up to 216 h (endpoint of the experiment) in triplicate; b) Inhibitory measured with calliper and expressed as mean followed by standard deviation of three technical replica one independent experiment.

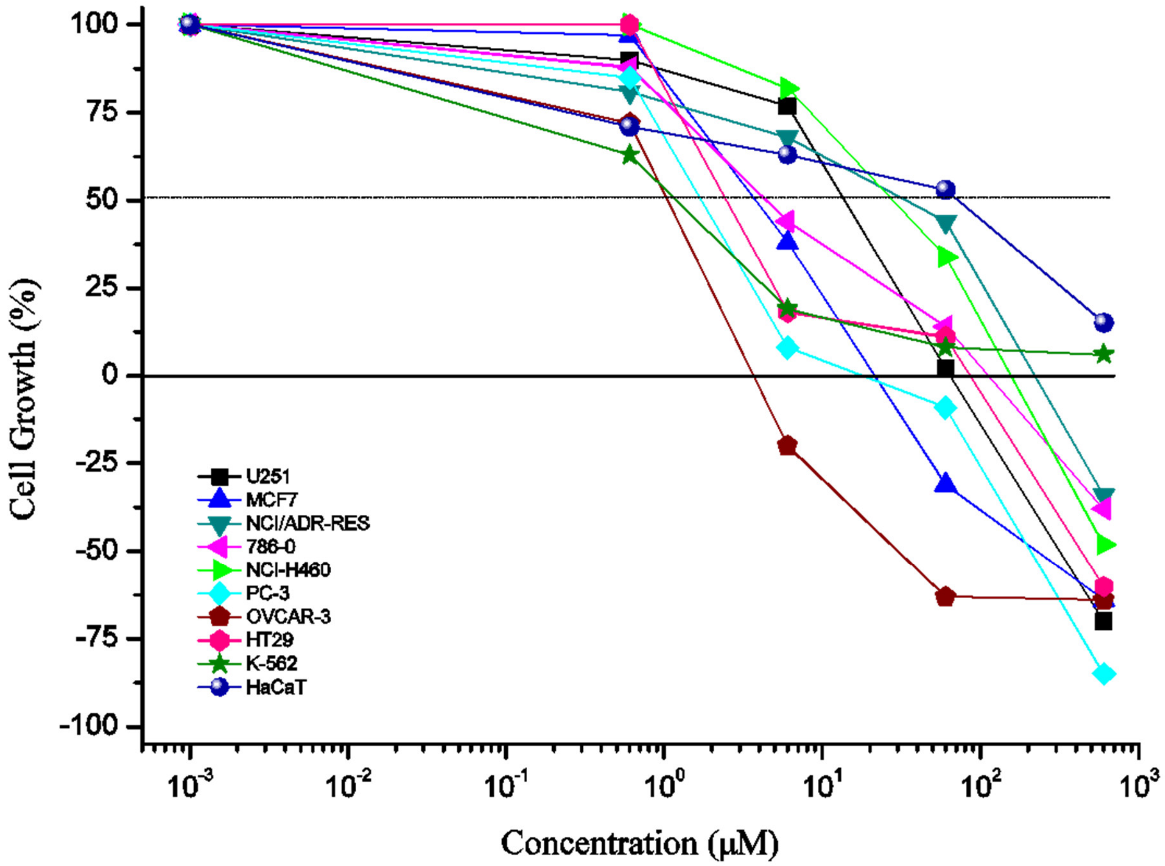

Cell Growth (%)
Concentration (µM)
U251
MCF7
NCI/ADR-RES
786-0
NCI-H460
PC-3
OVCAR-3
HT29
K-562
HaCaT

(A)

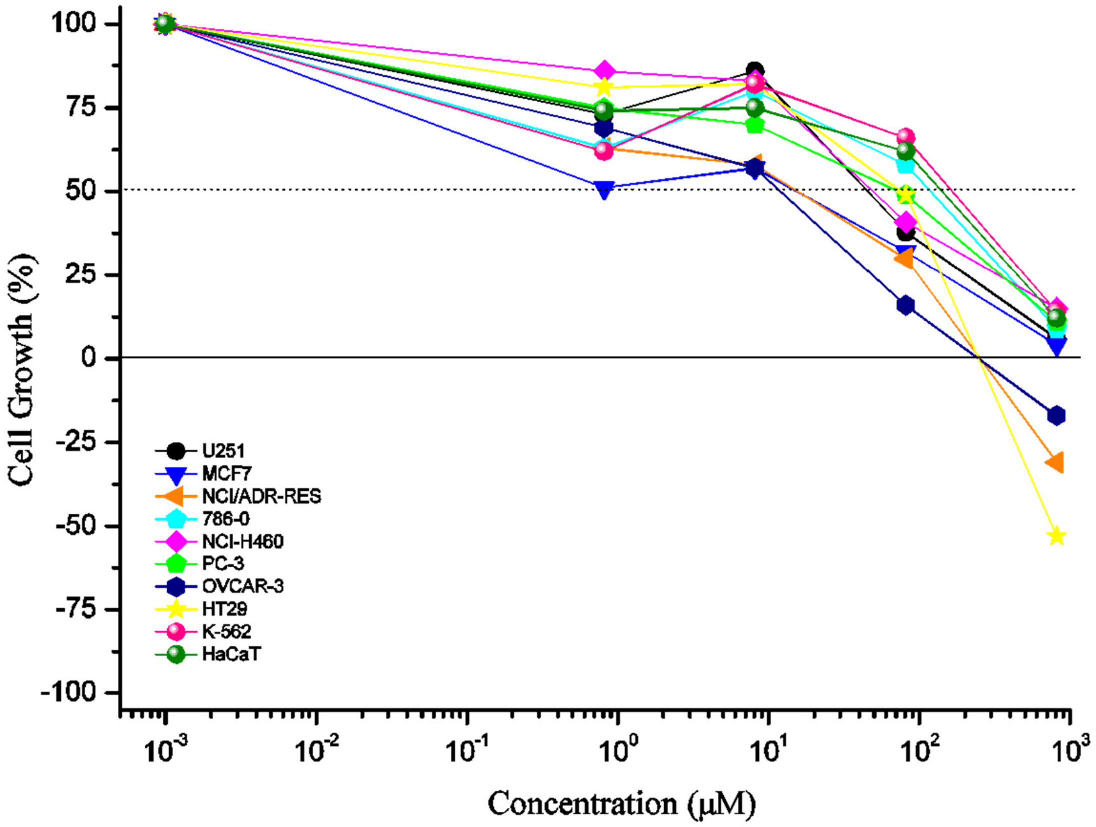

Cell Growth (%)
Concentration (µM)
U251
MCF7
NCI/ADR-RES
786-0
NCI-H460
PC-3
OVCAR-3
HT29
K-562
HaCaT

(B)

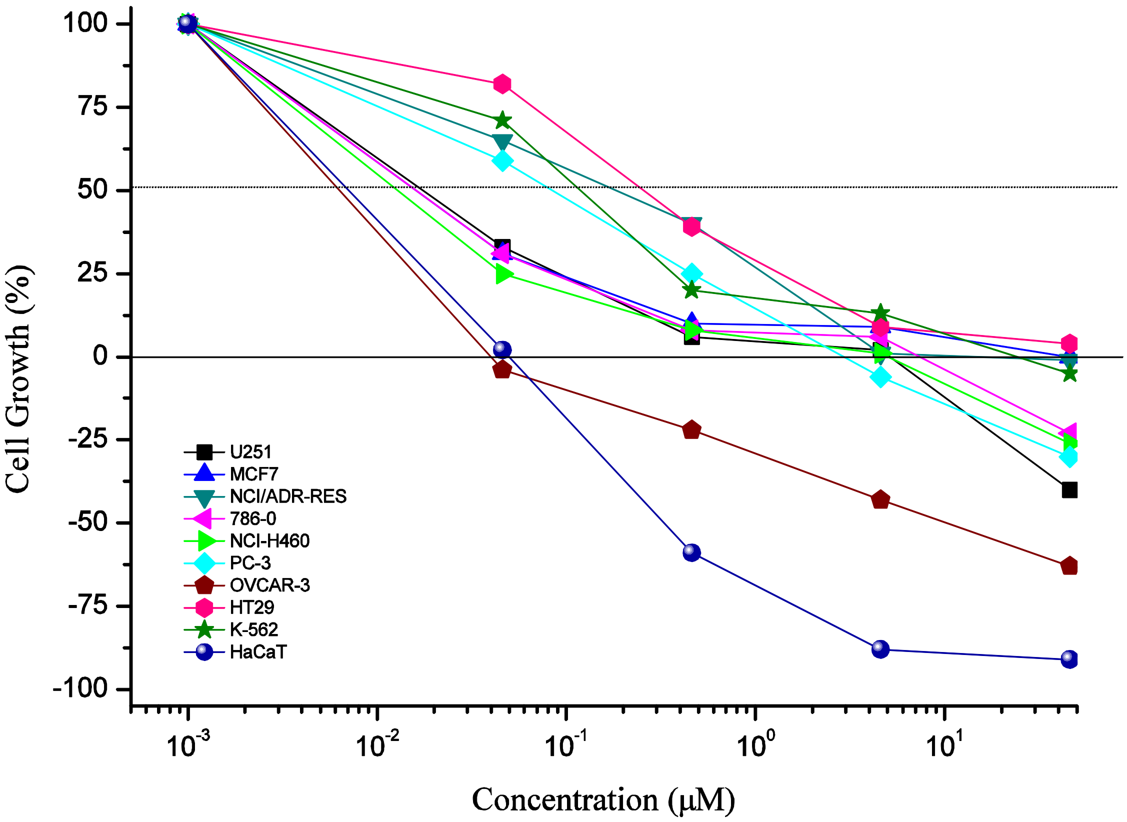

(C)

Figure S6. Antiproliferative profile of the AgNMS complex (**A**), nimesulide (**B**), and doxorubicin (**C**), against a human cell line panel after 48 h of exposure. Samples: **A)** AgNMS = silver nimesulide complex (concentration range: 0.25 – 250 μg/mL); **B)** NMS = nimesulide (concentration range: 0.25 – 250 μg/mL); **C)** doxorubicin (positive control, concentration range: 0.025 – 25 μg/mL); time exposure = 48 h.Human tumor cell lines: U251 (glioblastoma); MCF-7 (breast, adenocarcinoma); NCI-ADR/RES (multidrug-resistant high-grade ovarian serous adenocarcinoma); 786-0 (kidney, adeno-carcinoma); NCI-H460 (lung, non-small cell carcinoma); PC-3 (prostate, adenocarcinoma); OVCAR-03 (high-grade ovarian serous adenocarcinoma); HT29 (rectosigmoid adenocarcinoma); K562 (chronic myelogenous leukemia). Human non-tumor cell line: HaCaT (immortalized keratinocyte).

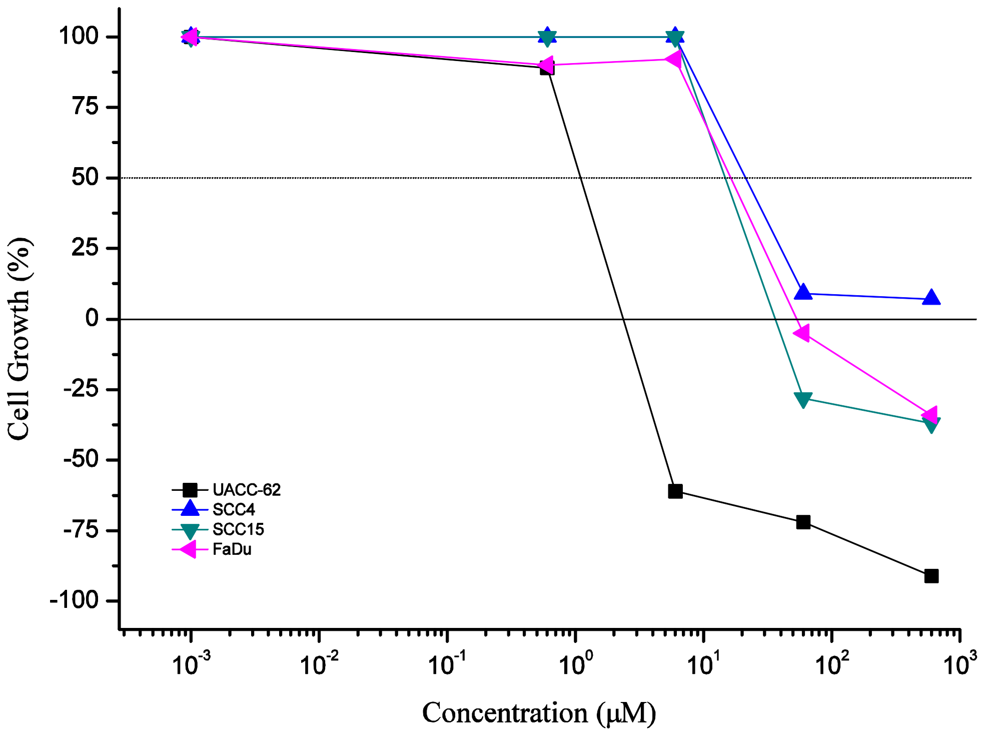

(A)

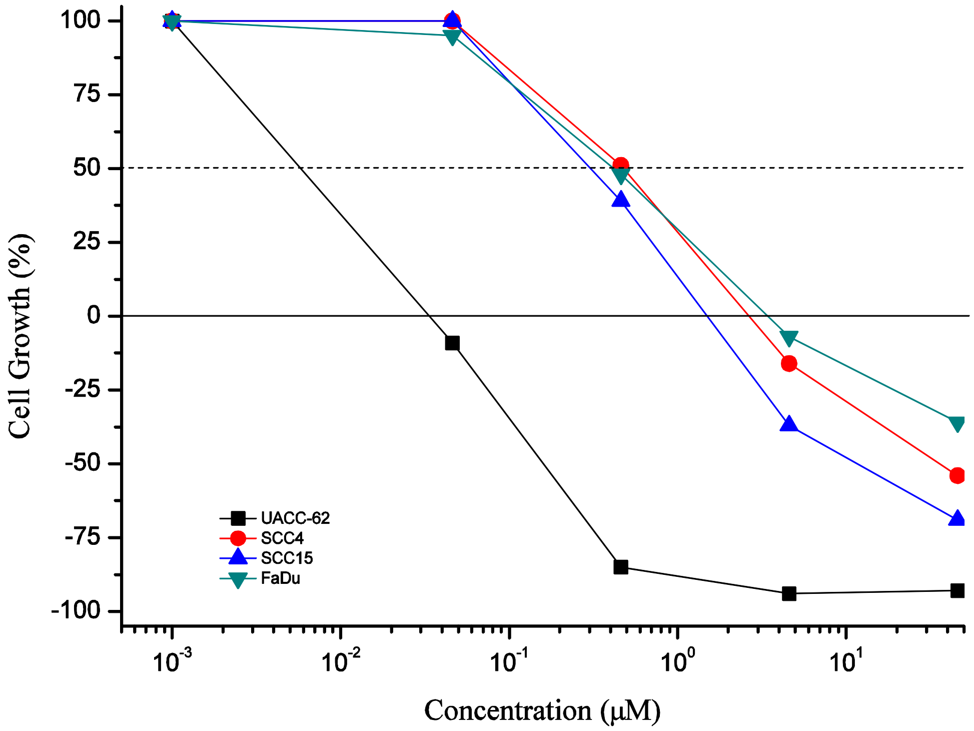

(B)

Figure S7. Antiproliferative profile of AgNMS complex (**A**),and doxorubicin (**B**) against squamous cell carcinoma and melanoma after 48 h of exposure. Samples: A) AgNMS.= silver nimesulide complex (concentration range: 0.25 – 250 μg/mL); B) doxorubicin (positive control, concentration range: 0.025–25 μg/mL). Time exposure = 48 h.Human tumor cell lines: SCC15 (squamous cell carcinoma of tongue); SCC4 (squamous cellcarcinoma of tongue); FaDu (squamous cell carcinoma of pharynx); UACC-62 (melanoma).
